# Hospital preparedness assessment for road traffic accidents with mass casualties: a cross-sectional study in Kurdistan Province, Iran

**DOI:** 10.1186/s12873-024-00981-4

**Published:** 2024-04-23

**Authors:** Arezoo Yari, Hamed Hassanzadeh, Kourosh Akhbari, Mohamad Esmaeil Motlagh, Khaled Rahmani, Yadolah Zarezadeh

**Affiliations:** 1 Social Determinants of Health Research Center, Research Institute for Health Development, Kurdistan University of Medical Sciences, Sanandaj, Iran; 2 Department of Health in Emergencies and Disasters, School of Medicine, Kurdistan University of Medical Sciences, Sanandaj, Iran; 3 Department of Emergency Medicine, Kosar Hospital, Kurdistan University of Medical Sciences, Sanandaj, Iran; 4 Department of Pediatrics, Ahvaz Jundishapur, University of Medical Sciences, Ahvaz, Iran; 5 Liver and Digestive Research Center, Research Institute for Health Development, Kurdistan University of Medical Sciences, Sanandaj, Iran; 6 Department of Medical Education, Medical School, Pasdaran Ave, Kurdistan University of Medical Sciences, 66186-34683 Sanandaj, Iran

**Keywords:** Hospital, Preparedness, Road traffic accidents, Mass casualties, Iran

## Abstract

**Background:**

Road traffic accidents (RTAs) are predicted to become the world’s seventh leading cause of death by 2030. Given the significant impact of RTAs on public health, effective hospital preparedness plays a pivotal role in managing and mitigating associated health and life-threatening issues. This study aims to meticulously evaluate the preparedness of selected hospitals in western Iran to handle road traffic accidents with mass casualties (RTAs-MC).

**Methods:**

The study employed a descriptive-analytical approach, utilizing a reliable and valid questionnaire to measure hospitals’ preparedness levels. Descriptive statistics (frequency distribution and mean) were utilized to provide an overview of the data, followed by analytical statistics (Spearman correlation test) to examine the relationship between hospital preparedness and its dimensions with the hospital profile. Data analysis, performed using SPSS software, categorized preparedness levels as weak, moderate, or high.

**Results:**

The study found that hospitals in Kurdistan province had a favorable preparedness level (70.30) to respond to RTAs-MC. The cooperation and coordination domain had the highest preparedness level (98.75), while the human resource management (59.44) and training and exercise (54.00) domains had the lowest preparedness levels. The analysis revealed a significant relationship between hospital preparedness and hospital profile, including factors such as hospital specialty, number of beds, ambulances, staff, and specialized personnel, such as emergency medicine specialists.

**Conclusion:**

Enhancing preparedness for RTAs-MC necessitates developing response plans to improve hospital profile, considering the region’s geographic and topographic features, utilizing past experiences and lessons learned, implementing of Hospital Incident Command System (HICS), providing medical infrastructure and equipment, establishing communication channels, promoting cooperation and coordination, and creating training and exercise programs.

**Supplementary Information:**

The online version contains supplementary material available at 10.1186/s12873-024-00981-4.

## Background

Road traffic accidents (RTAs) are one of the most significant public health issues in the world today [1, 2], with considerable adverse effects on the health of individuals and communities [3]. Every year, 1.35 million people are killed or disabled due to traffic accidents worldwide [1]. Unfortunately, traffic accidents affect mainly the lives and health of children, youth, and individuals of working age. Besides the medical costs for the injured and disabled, traffic accidents have significant economic consequences for societies [3]. The health effects of RTAs are of even greater importance in developing countries [2]. In fact, in 2019, 93% of deaths related to RTAs occurred in low- and middle-income countries, resulting in approximately 1.3 million deaths [1].

The growth of this problem suggests the possibility of an increase in health problems, mortality, and medical issues resulting from it in the coming years. According to estimations, by 2030, RTAs will be the seventh leading cause of death globally [1]. Even if appropriate actions are not taken, it may become one of the top five leading causes of death worldwide [2, 4]. In addition, the non-fatal consequences and injuries resulting from traffic accidents can lead to a rise in general medical problems in different regions of the world [2]. The consequences of traffic accidents occupy hospital beds, increase the need for financial, medical, and public health resources for the treatment of traffic injury victims, and ultimately endanger the capacity of healthcare systems [3].

Hospitals play a vital role in providing medical care and first aid facilities on busy roads and streets during RTAs [5]. Since RTAs can lead to the death or injury of one or more individuals [2], the first place where traffic injury victims go or need services is hospitals and healthcare centers [6]. In other words, hospitals provide ambulances and trained healthcare personnel to transport and transfer injured individuals. In addition to providing emergency care to traffic injury victims at the time of the accident, the healthcare system also provides rehabilitation services for these victims [5].

The increase in the burden of hospital patients and the need for timely and effective provision of medical services by hospitals highlight the importance of hospital preparedness during RTAs, especially those resulting in mass casualty incidents (MCIs) [6]. In addition, to improve preparedness for emergencies and in line with international commitments, countries are encouraged to improve their healthcare systems and strengthen national capacity for emergency health management, crisis response, and flexibility in healthcare systems [7]. Considering the role of hospitals as vital components of the healthcare system in responding to disasters and emergencies to ensure service continuity and effective delivery [8, 9], the World Health Organization emphasizes the importance of hospital preparedness and response capacity [9–11]. With the provision of hospital preparedness plans and crisis management strategies, significant effects can be achieved in reducing mortality rates [12]. According to the Hospital Safety Index (HSI) defined by the World Health Organization (WHO), the most important factor in hospital performance is “having preparedness plans to deal with emergency conditions and unexpected events.” In this regard, the evaluation of hospital preparedness can indicate the necessary gap in readiness during emergencies and the current level of preparedness [6].

Although RTAs have received attention from health institutions in today’s world [1], unfortunately, statistics indicate that despite the United Nations’ Decade of Action for Road Safety (2011–2020) coming to an end, countries have made much less progress in reducing traffic-related injuries compared to other health areas [13]. In addition, studies indicate that hospitals have insufficient preparedness to respond to disasters and may face various operational difficulties and shortcomings when responding to accidents [9, 14]. According to research on assessment of hospital preparedness in Kampala, the main weakness in responding to RTAs in the pre-hospitalization sector is the lack of a pre-developed plan [15].

RTAs are the fourth leading cause of death in Iran [16] and the largest cause of years of life lost (YLL) in the country [17]. According to the WHO estimate in Iran, the mortality rate due to RTAs is 20.5 per 100,000 people [18]. Studies in this country have shown that RTAs impose a significant economic burden on Iran’s gross domestic product per capita and health expenses per capita [3]. The focus of this research is on Kurdistan province, located in west of Iran, with an area of 28,235 square kilometers [7] and a population of nearly one and a half million people [19], which accounts for about 1.7% of the country’s area [7]. The topography, geographic and ethnic diversity, and border location of this province have made it susceptible to various natural and human disasters [7, 20].

The intricate network of roads within the province is vital for connectivity of the northern, southern, and western regions of Iran. This network is intricately woven into this province’s mountainous terrain and presents a significant risk factor for RTAs [21]. The challenging topography, coupled with well-traveled roads, amplifies the vulnerability of Kurdistan province to accidents. Alarming statistics underscore the severity of the situation, revealing a considerably higher number of casualties from RTAs in Kurdistan province compared to the national average in Iran [22–24].

These distinctive characteristics not only describe the Kurdistan province but also underscore the underlying vulnerability of the region. The confluence of mountains, diverse landscapes, and border adjacency forms a complex tapestry that demands specialized attention, particularly in the context of disaster preparedness and response. Understanding and addressing these intricacies become paramount for effective regional governance and, specifically, in the context of this study, for comprehending the challenges and nuances of hospital preparedness in the face of road traffic accidents. This reality emphasizes the critical need for heightened hospital attention and preparedness to effectively address and mitigate the impact of these incidents on the local community.

## Methods

In this study, which was based on the results of a thesis by a medical student at Kurdistan University of Medical Sciences, the level of hospital preparedness in Kurdistan province for RTAs-MC in the year 2022 was examined.

### Sample size and sampling method

The sampling method in this study was convenience sampling and all hospitals in the geographical area of Kurdistan province were included in the study through a census. Hospitals that were not willing to cooperate and participate in the study were excluded from the study.

### Study design and setting

**Fig. 1:**
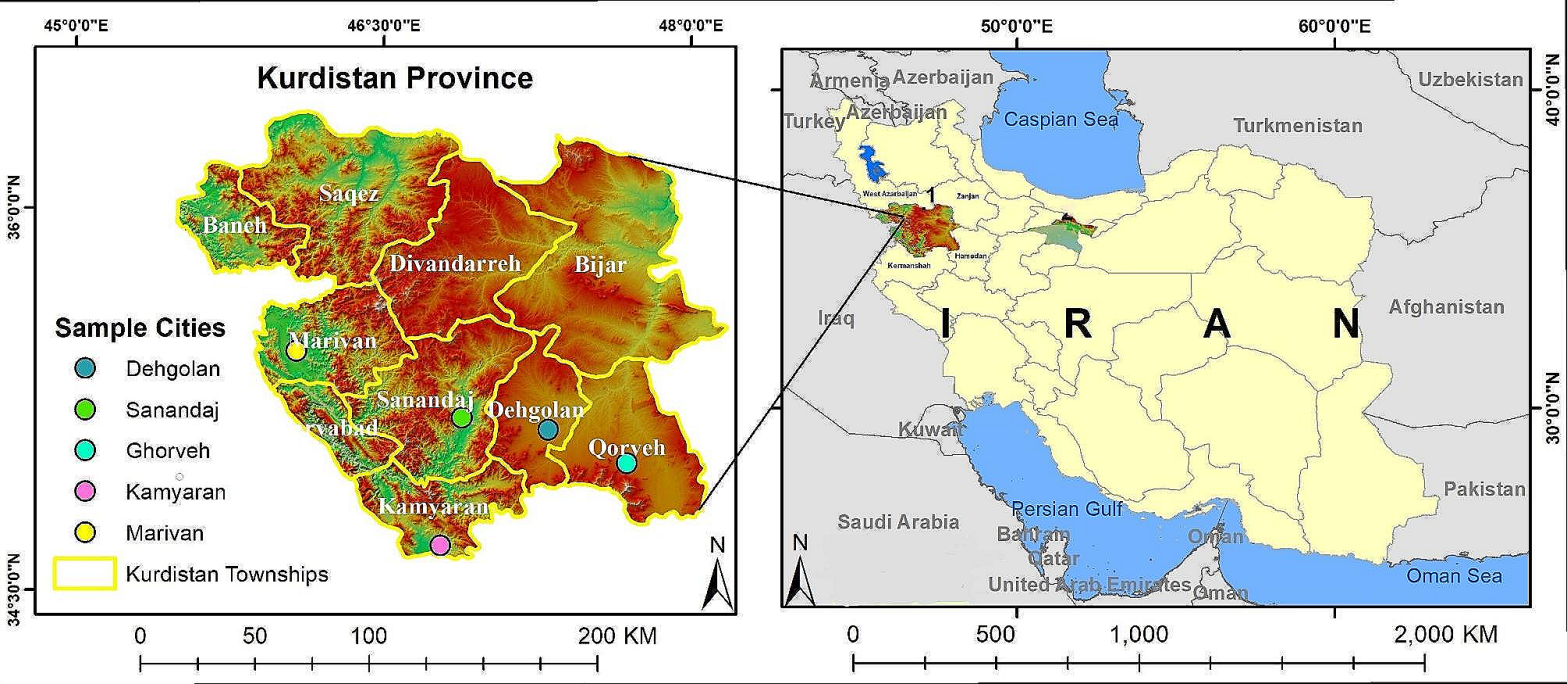
The Location of Kurdistan province and its cities in Iran. This map shows Iran?s administrative and political borders with neighboring countries. Additionally, it displays the borders of neighboring provinces of Kurdistan and its bordering position with Iraq. The marked regions represent Kurdistan rovince and its cities, where the research was conducted. The authors have been inspired by the reference below and have made some modifications to create the map they used: Reference: Ramazani R, Yari A, Heydari A, Hanafi-Bojd AA, Soltani A, Rostami S, Ostadtaghizadeh A. War, displacement, and the best location for temporary sheltering: a qualitative study. BMC Public Health. 2022 Nov 11;22 [1]:2066

This research comprised a cross-sectional study with descriptive-analytical approach, conducted in Kurdistan Province (Fig. 1).

The road network of the province provides it with connections to West Azerbaijan and Zanjan provinces to the north, Kermanshah province to the south, and Hamedan and Zanjan provinces to the east [25]. In addition to the domestic road network, this province shares a border with Iraq to the west. It has ten counties [26] and is a mountainous region with unique geographical conditions, highlands, cold and snowy weather, and even icy conditions in the winter season [25].

The data for this study were systematically collected from hospitals within Kurdistan Province, encompassing various types of healthcare facilities, including government, private, social security, or military institutions. The inclusion criteria were hospitals located within the geographical boundaries of Kurdistan Province that expressed willingness to collaborate and provide the necessary information for the study. Government hospitals in Kurdistan Province, under the supervision of Kurdistan University of Medical Sciences, were included, irrespective of whether they were educational or non-educational.

The variability in the number and types of specialties offered by the hospitals in the province was taken into account, including single-specialty, general, specialty, or multiple specialties. Exclusion criteria were applied to hospitals that did not cooperate or were unwilling to share essential information for the research. After a thorough consideration of these factors, a total of 20 hospitals meeting the specified criteria were selected for inclusion in this study. This inclusive selection process ensures a comprehensive representation of the hospital landscape in the region, enhancing the transparency of our methodology. The classification of hospitals in Iran is based on their organizational affiliation, and hospitals are categorized and named accordingly. The main categories are as follows: Government General Hospitals (GGH): hospitals owned and supervised by the Ministry of Health and Medical Education, which provide free medical services to patients. Government– Educational Hospitals (GEH): hospitals that owned and governed by universities of medical sciences and other educational institutions, and also function as research and educational centers. Private Hospitals (PH): hospitals owned by private companies, legal entities, or charities that provide medical services to patients with a fee. Military Hospitals (MH): hospitals under the supervision of the Ministry of Defense, which provide free medical services to military personnel and their families. Social Security Hospitals (SSH): hospitals owned by the Ministry of Cooperatives, Labor, and Social Welfare, which provide free medical services to members of the social security system [27, 28]. In this study, hospitals were classified into three categories based on their specialty: general hospitals, specialized hospitals, and tertiary hospitals. General hospitals provide a wide range of medical services and treat various types of diseases. Specialized hospitals are dedicated to providing specialized medical care, such as cancer or orthopedic treatment. Tertiary hospitals deliver the highest level of medical care to general population.

### Data collection

The data were collected using an assessment tool prepared by Hamid Safarpour and colleagues [6], specifically designed to evaluate hospital preparedness for RTAs-MC in the context of Iran. This tool has an acceptable validity and reliability (Kappa coefficient = 0.89; CVR: 0.98; CVI: 0.97). This tool measures nine different domains of hospital preparedness for RTAs-MC, including command and control, infrastructure, medical equipment, communication and information systems, capacity building, triage and medical services, safety and security, human resources, coordination, training, and exercises [6]. In addition to the nine domains mentioned above, this checklist also examines the resources and specialized equipment for managing trauma patients, which is not reported in this article. The decision to employ this native version ensures that the assessment tool is culturally appropriate and tailored to the specific challenges and nuances of the Iranian healthcare system.

The questions in the checklist assessing the different domains of hospital preparedness were designed on a Likert scale with “yes,” “somewhat,” and “no” as the possible answers. Questions with “no” received a score of one, questions with “somewhat” received a score of two, and questions with “yes” received a score of three. Finally, the level of hospital preparedness was categorized into three groups: weak (less than 34%), moderate (34–66%), and high (more than 67%). The checklist for assessing hospital preparedness for RTAs-MC had 78 questions related to the relevant domains. The command and control domain had nine questions, the infrastructure and medical equipment domain had fifteen questions, the communication and information systems domain had eight questions, the surge capacity domain had twelve questions, the triage and medical services domain had 13 questions, the safety and security domain had five questions, the human resources domain had nine questions, the coordination domain had two questions, and the training and exercises domain had five questions. In addition to the above checklist, the research team added 13 researcher-made questions to the questionnaire regarding the general characteristics of the hospitals. Ultimately, the questionnaire had 91 questions.

In this study, hospital profiles were collected, including information related to the type of hospital, hospital specialty, number of hospital beds and their occupancy rate, annual patient admissions, number of staff, and the presence of trauma departments, emergency medicine specialists, and disaster experts.

### Data analysis

The collected data was analyzed using SPSS 22 statistical software. Descriptive statistics (frequency distribution and mean) and analytical statistics (Spearman correlation test) were used to examine the relationship between hospital preparedness and its dimensions with hospital profile. Additionally, to determine the preparedness status of hospitals for RTAs-MC, the obtained scores were calculated as percentages and the classification model of the safety status of Iranian hospitals called the Farsi Hospital Safety Index (FHSI) was considered. The hospital preparedness status in RTAs-MC was classified into three levels: poor (less than 34%), moderate (66 − 34%), and high (more than 66%) [9, 11].

## Results

From the initially considered 21 hospitals in Kurdistan Province, 20 hospitals actively participated in our study. Only one private hospital declined to collaborate in this research. Descriptive analysis of the characteristics of the hospitals surveyed showed that 60% of the hospitals in Kurdistan province were government general hospitals and, in terms of specialization.

In terms of the number of approved beds, the majority (85%) of hospitals in this province have less than or equal to 300 approved beds and have less than or equal to 200 active beds. Furthermore, 80% of hospitals have less than or equal to 30 emergency beds.

The majority of hospitals in Kurdistan province (55%) have between 60,000 and 150,000 annual admissions. In terms of the total number of employees, over 50% of the hospitals in the province have 200–400 staff. Most hospitals in the province (60%) have between 300 − 100 clinical staff. Almost half (45%) of the hospitals in the province have 50–100 non-clinical staff. 55% of hospitals in this province have 3 or 4 ambulances. All hospitals in the province have an emergency specialist, and only one hospital has a trauma center. Additionally, the majority of hospitals in the province (80%) do not have an emergency medicine specialist (Table 1).

**Table 1:** General profile of studied Hospitals in Kurdistan, Iran

City	Hospital	Type	Specialty	Bed Count (n)	Active Bed Count (n)	Emergency Bed Count (n)	Bed occupancy (%)	Annual admission (n)	Employee Count (n)	Clinical employee (n)	Non-clinical employee (n)	Ambulance (n)	Emergency Preparedness Coordinator	Trauma Center	Emergency Medicine Specialist
Sanandaj	A	GEH	Tertiary	540	441	60	61%	167,382	1152	819	333	3	Yes	Yes	Yes
	B	GEH	Tertiary	536	338	41	69.5%	270,392	916	603	313	4	Yes	No	No
	C	GEH	Specialized	130	119	6	80%	1607	186	95	91	1	Yes	No	No
	D	GEH	Tertiary	518	345	45	82%	100,691	836	504	332	4	Yes	No	Yes
	E	SSH	Tertiary	160	147	10	58%	215,610	400	248	152	4	Yes	No	Yes
	F	MH	General	64	45	4	41%	5306	103	65	38	2	Yes	No	No
	G	PH	General	50	50	4	38%	33,614	128	79	49	1	Yes	No	No
Bijar	H	GGH	General	118	108	21	50%	33,614	316	256	160	4	Yes	No	No
Marivan	I	GGH	Tertiary	134	120	14	94%	97,286	377	277	100	6	Yes	No	No
	J	GGH	General	100	85	13	85%	126,800	272	184	88	1	Yes	No	No
Saqez	K	GGH	General	296	116	32	70%	116,498	560	373	187	4	Yes	No	No
	L	GGH	Tertiary	235	165	20	80%	106,812	331	236	95	5	Yes	No	Yes
	M	SSH	General	52	52	7	76%	15,871	209	112	97	4	Yes	No	No
Dehgolan	N	GGH	General	42	32	8	67%	135,984	192	68	124	3	Yes	No	No
Divandarh	O	GGH	General	91	74	13	40.78%	104,853	303	196	107	3	Yes	No	No
Baneh	P	GGH	General	153	96	12	75%	83,759	286	198	88	7	Yes	No	No
	Q	GGH	Tertiary	64	56	21	38.2%	28,083	223	130	93	3	Yes	No	No
Kamyaran	R	GGH	General	101	77	10	60%	109,568	351	267	84	4	Yes	No	No
Gorveh	S	GGH	General	227	140	26	63.11	121,311	418	235	183	5	Yes	No	No
Sarvabada	T	GGH	General	32	32	10	56%	31,552	132	76	56	2	Yes	No	No

*GEH*: Government– Educational Hospital, *GGH*: Government General Hospital, *SSH*: Social Security Hospital, *MH*: Military Hospital, *PH*: Private Hospital

The results of hospital preparedness assessment, based on overall preparedness, showed that the overall preparedness level of hospitals in Kurdistan province was 70.30% in RTAs-MC. Out of the seven hospitals studied in Sanandaj, only two hospitals had a moderate level of preparedness. Hospitals in the cities of Bijar (65.06), Kamyaran (65.66), Dehgolan (60.84) and Sarvabad (53.61) had a moderate level of preparedness. Additionally, two other hospitals in Baneh and Marivan had a moderate level of preparedness for RTAs-MC. The lowest level of preparedness (48.80%) and the highest level of preparedness (89.76%) were related to hospitals in the city of Sanandaj. Furthermore, the results of the hospital preparedness analysis based on the studied domains showed that the hospitals’ preparedness level in all domains except for two domains, namely human resources management (59.44) and training and exercises (54.00), was high. In fact, the lowest level of preparedness was related to the domain of training and exercises, while the highest level of preparedness was related to the domain of collaboration and coordination (98.75) (Table 2).

**Table 2:** Levels and scores of preparedness dimensions of Hospitals for response to RTAs-MC in Kurdistan, Iran

City	Hospitals Dimensions	Command and Control	Infrastructure and medical equipment	Communication and information systems	Surge capacity	Triage and medical services	Safety and security	Human resources management	Coordination and cooperation	Training and exercise	Total Preparedness	Total Preparedness level
Sanandaj	A	88.89	92.50	83.33	95.83	100.00	80.00	72.22	100.00	70.00	89.76	High
	B	77.78	82.50	61.11	87.50	73.08	60.00	55.56	100.00	30.00	72.89	High
	C	50.00	50.00	44.44	50.00	42.31	90.00	38.89	100.00	10.00	48.80	Moderate
	D	77.78	80.00	66.67	91.67	80.77	80.00	66.67	100.00	40.00	77.71	High
	E	66.67	75.00	77.78	83.33	92.31	100.00	83.33	100.00	80.00	82.53	High
	F	55.56	57.50	50.00	66.67	65.38	100.00	66.67	100.00	60.00	64.46	Moderate
	G	72.22	80.00	66.67	91.67	76.92	100.00	66.67	100.00	50.00	78.31	High
Bijar	H	61.11	62.50	55.56	70.83	73.08	70.00	55.56	100.00	50.00	65.06	Moderate
Marivan	I	83.33	55.00	72.22	75.00	69.23	60.00	55.56	100.00	60.00	67.47	High
	J	55.56	62.50	72.22	58.33	76.92	90.00	50.00	100.00	40.00	65.06	Moderate
Saqez	K	72.22	77.50	77.78	70.83	76.92	100.00	61.11	100.00	70.00	76.51	High
	L	88.89	70.00	77.78	75.00	73.08	100.00	66.67	100.00	80.00	80.12	High
	M	66.67	65.00	72.22	66.67	84.62	100.00	55.56	100.00	70.00	72.29	High
Dehgolan	N	55.56	65.00	55.56	45.83	69.23	80.00	50.00	100.00	50.00	60.84	Moderate
Divandarh	O	66.67	77.50	77.78	83.33	73.08	100.00	61.11	100.00	70.00	77.11	High
Baneh	P	83.33	65.00	83.33	70.83	84.63	80.00	55.56	100.00	80.00	75.30	High
	Q	50.00	70.00	61.11	66.67	65.38	100.00	50.00	100.00	30.00	64.46	Moderate
Kamyaran	R	83.33	52.50	55.56	66.67	80.77	80.00	55.56	75.00	50.00	65.66	Moderate
Gorveh	S	55.56	70.00	55.56	70.83	69.23	90.00	66.67	100.00	50.00	68.07	High
Sarvabada	T	38.89	37.50	66.67	58.33	65.38	60.00	55.56	100.00	40.00	53.61	Moderate
Mean		67.50	67.87	66.67	72.29	74.61	86.00	59.44	98.75	54.00	70.30	High
preparedness level in studied dimensions		High	High	High	High	High	High	Moderate	High	Moderate	High	

The Spearman correlation test was employed to assess the relationship between hospital preparedness and its dimensions with the hospital profile. This non-parametric measure of statistical dependence is particularly suitable for evaluating the strength and direction of monotonic relationships between variables, especially when dealing with ordinal or non-normally distributed data. In the context of this study, the Spearman correlation test was utilized to explore the association between different dimensions of hospital preparedness and some relevant hospital characteristics.

The analytical analysis of the relationship between hospital characteristics and preparedness level for RTAs-MC, as well as their domains, showed that while the type of hospital had no significant relationship with its preparedness level and domains for RTAs-MC, the hospital’s specialty had a significant and positive relationship with its preparedness level based on Infrastructure and medical equipment (P-value < 0.05, as shown in Table 3).

**Table 3:** Relationship between Hospital profile and preparedness for response to RTAs-MC in Kurdistan, Iran

Hospital Profile			Command and Control	Infrastructure and medical equipment	Communication and information systems	Surge capacity	Triage and medical services	Safety and security	Human resources management	Coordination and cooperation	Training and exercise	Total Preparedness
		**Number (%)**	Spearman’s rho (P-value)	Spearman’s rho (P-value)	Spearman’s rho (P-value)	Spearman’s rho (P-value)	Spearman’s rho (P-value)	Spearman’s rho (P-value)	Spearman’s rho (P-value)	Spearman’s rho (P-value)	Spearman’s rho (P-value)	Spearman’s rho (P-value)
Type	GEH	4 [20]	0.271 (0.248)	0.187 (0.430)	0.110 (0.643)	0.197 (0.406)	0.061 (0.797)	-0.110 (0.644)	-0.495* **(0.026)**	-	-0.015 (0.950)	-0.271 (0.248)
	GGH	12 [29]										
	SSH	2 [10]										
	MH	1 [5]										
	PH	1 [5]										
Specialty	General	12 [29]	0.308 (0.186)	0.463* **(0.040)**	0.092 (0.698)	0.239 (0.310)	0.025 (0.916)	-0.258 (0.281)	0.295 (0.206)	-	-0.136 (0.568)	0.308 (0.186)
	Specialized	1 [5]										
	Tertiary	7 [30]										
Approved bed Count (n)	< 50	4 [20]	0.444* **(0.026)**	0.480* **(0.032)**	0.054 (0.820)	0.422 (0.064)	0.422 (0.064)	-0.037 (0.876)	0.242 (0.304)	-	0.134 (0.575)	0.526* **(0.017)**
	50–100	4 [20]										
	100–200	6 [31]										
	200–300	3 [15]										
	> 300	3 [15]										
Active Bed Count (n)	< 50	4 [20]	0.360 (0.119)	0.461* **(0.041)**	0.055 (0.817)	0.350 (0.130)	0.350 (0.130)	-0.101 0.671	0.218 (0.356)	-	0.046 (0.849)	0.480* **(0.032)**
	50–100	6 [31]										
	100–200	7 [30]										
	200–300	0 (0)										
	> 300	3 [15]										
Emergency Bed Count (n)	< 10	8 [32]	0.185 (0.434)	0.545* **(0.013)**	0.046 (0.846)	0.397 (0.083)	0.307 (0.189)	-0.064 0.790	0.057 (0.811)	-	0.008 (0.974)	0.380 (0.098)
	10–20	5 [25]										
	20–30	3 [15]										
	> 30	4 [20]										
Bed occupancy (%)	< 50	5 [25]	0.253 (0.283)	-0.284 (0.225)	0.383 (0.095)	-0.229 (0.331)	0.229 (0.331)	-0.192 (0.416)	-0.115 (0.628)	-	0.139 (0.558)	0.253 (0.283)
	50–70	8 [32]										
	70–90	6 [31]										
	> 90	1 [5]										
Annual admission (n)	> 20,000	3 [15]	0.428 (0.060)	0.419 (0.066)	0.146 (0.539)	0.262 (0.264)	0.667* **(0.001)**	-0.133 (0.575)	0.140 (0.556)	-	0.275 (0.241)	0.428 (0.060)
	20,000–60,000	3 [15]										
	60,000–150,000	11 [33]										
	150,000–200,000	1 [5]										
	> 200,000	2 [10]										
Total Employee (n)	> 200	5 [25]	0.459* **(0.042)**	0.562* **(0.010)**	0.153 (0.520)	0.527* **(0.017)**	0.527* **(0.017)**	-0.026 (0.913)	0.157 (0.508)	-	0.208 (0.379)	0.574* **(0.008)**
	200–400	10 [34]										
	400–600	2 [10]										
	600–800	0 (0)										
	> 800	3 [15]										
Clinical employee (n)	> 100	5 [25]	0.529* **(0.016)**	0.510* **(0.022)**	0.137 (0.565)	0.559* **(0.010)**	0.559* **(0.010)**	-0.100 (0.674)	0.206 (0.383)	-	0.212 (0.370)	0.529* **(0.016)**
	100–200	5 [25]										
	200–300	6 [31]										
	300–500	1 [5]										
	> 500	3 [15]										
Non-clinical employee (n)	> 50	2 [10]	0.205 (0.385)	0.485* **(0.030)**	-0.019 (0.938)	0.194 (0.411)	0.446* **(0.049)**	-0.038 (0.872)	0.106 (0.658)	-	0.131 (0.582)	0.364 (0.114)
	50–100	9 [35]										
	100–150	2 [10]										
	150–200	4 [20]										
	> 200	3 [15]										
Ambulance (n)	1	3 [15]	0.509* **(0.022)**	0.116 (0.627)	0.109 (0.647)	0.580* **(0.007)**	0.524* **(0.018)**	-0.125 (0.600)	0.028 (0.907)	-	0.350 (0.120)	0.546* **(0.013)**
	2	2 [10]										
	3	4 [20]										
	4	7 [30]										
	5	2 [10]										
	6	1 [5]										
	7	1 [5]										
Emergency Preparedness Coordinator	Yes	20 (100)	-	-	-	-	-	-	-	-	-	-
	No	0 (0)										
Trauma Center	Yes	1 [5]	-0.187 (0.479)	-0.229 (0.331)	-0.187 (0.429)	-0.115 (0.630)	-0.115 (0.630)	-0.096 (0.686)	-0.313 (0.180)	-	-0.284 (0.225)	-0.187 (0.429)
	No	19 (95)										
Emergency Medicine Specialist	Yes	4 [20]	-0.407 (0.074)	-0.500* **(0.025)**	-0.408 (0.074)	-0.250 (0.288)	-0.250 (0.288	-0.210 (0.374)	-0.681* **(0.001)**	-	-0.416 (0.068)	-0.408 (0.074)
	No	16 (80)										

Additionally, the analytical analysis indicated a positive and significant relationship between the approved and active bed numbers of hospitals and the overall hospital preparedness level for RTAs-MC, while this relationship was not observed with the number of emergency beds. Moreover, the approved bed numbers of hospitals were only positively and significantly related to the domains of command and control and Infrastructure and medical equipment, while the active bed numbers and the number of emergency beds were only positively and significantly related to the domain of Infrastructure and medical equipment. Furthermore, the results of the analytical analysis revealed a significant and positive relationship between the annual admission rate of the hospital and its preparedness level in the triage and medical services domains (P-value < 0.05, as shown in Table 3).

In addition to the above results, the analytical assessment of the relationship between the number of hospital employees and hospital preparedness for traffic accidents revealed the following significant correlations. The total number of employees, clinical employees, and overall hospital preparedness level for RTAs-MC in the command and control and surge capacity domains had a significant and positive correlation, while this association was not observed with the number of non-clinical employees. Moreover, the total number of employees, clinical employees, and non-clinical employees had a significant and positive correlation with the triage and medical services domains, as well as the infrastructure and medical equipment domain. The number of ambulances had a significant and positive correlation with the overall hospital preparedness level for traffic accidents in the command and control, surge capacity, and triage and medical services domains. Furthermore, the analytical results indicated significant and inverse correlation between the no presence of emergency medicine specialists and the infrastructure and medical equipment domain, and human resources domains (P-value < 0.05, as shown in Table 3).

## Discussion

Most studies related to the assessment of hospitals’ preparedness for RTAs have mainly focused on describing the level of preparedness and its domains. However, this study not only describes the hospital’s preparedness status for RTAs-MC and its domains, but also analyzes its correlation with hospital profile using Spearman correlation analysis. As a result, this study takes a different approach from previous studies in its descriptive approach. Furthermore, this study was conducted in Kurdistan province, Iran, which is susceptible to RTAs-MC due to its unique topography and geographical location.

Previous studies have reported undesirable hospitals’ preparedness in border provinces [31] and Kurdistan province in Iran [36] for emergencies and disasters. However, despite the moderate level of preparedness in some hospitals in Kurdistan province for RTAs-MC, overall preparedness was desirable, which was consistent with the study by Mohammadi et al. in Kermanshah [37]. In a study by Yousefian et al. in Iran, which used a similar tool to assess hospitals’ preparedness for RTAs-MC, the results indicated a moderate hospitals’ preparedness [9]. Moreover, the results of a meta-analysis study of 181 hospitals [38] and a systematic review study [36] in Iran have shown that the Iranian Hospital Disaster Preparedness (HDP) is moderate. However, some Iranian hospitals have a low HDP [12, 36, 39, 30]. It is possible that the higher preparedness in the present study may be due to the timing of the study, which was conducted after the COVID-19 crisis, meaning that the occurrence of this crisis may have improved hospitals’ preparedness in the province.

Comparison studies have shown that Iranian HDP is lower than that of developed countries [40]. Among these countries with higher HDP are the UK, Lithuania, and Luxembourg, as reported in a study in Europe where an acceptable preparedness was reported in a significant number of European countries [41]. However, a study in the Netherlands indicated insufficient or inadequate HDP [42]. The difference in findings could be attributed to variations in the assessment tools, location, and timing of the studies. Additionally, the present study only examined hospital preparedness for RTAs-MC, while most of the mentioned studies examined preparedness for all types of emergencies and disasters. Therefore, it is crucial for each region or country, considering its unique geographical, cultural, and social characteristics, to conduct tailored assessments of hospital preparedness for RTAs-MC. This underscores the importance of developing and implementing region-specific strategies to elevate hospital preparedness. As hospitals in different areas face diverse challenges, addressing these challenges will not only benefit the preparedness for RTAs-MC but will also contribute to better overall disaster preparedness.

Command and control are fundamental principles of planning for managing major incidents [43]. In fact, the Hospital Incident Command System (HICS) can lead to a significant improvement in managing the response to major incidents and disasters [32]. It helps to ensure the continuity of health delivery services in emergency situations and can be used as a management system for organizing personnel, facilities, equipment, and communications [44]. Therefore, establishing a standardized HICS will lead to regular and accurate organization of human resources and management tasks, the development of unity of command, and the improvement of incident management in hospitals [44]. The level of hospital preparedness assessed in the present study was desirable in terms of command and control, which is consistent with some internal studies in Iran [9, 45]. The highest score for command and control was related to two hospitals, A and L, located in the cities of Sanandaj and Saqez, respectively. Both of these hospitals are tertiary hospitals with the highest level of service delivery, and Sanandaj is the capital of the province [26], with Hospital A being the largest hospital in the province. Although many hospitals have established an HICS, attention to the factors that influence its success, efficiency, and qualitative aspects has been overlooked [46]. Therefore, having a response plan that is appropriate to the conditions and situation of each hospital [9, 47] and paying attention to allocating sufficient resources should be considered in the command-and-control domain [9].

To enhance coordination and provide vital information among the incident command teams, the use of advanced communication technologies is necessary [46]. Coordination plays a crucial role in improving the efficiency of the system in managing emergencies [46, 35]. In response to MCI, preparedness in the field of information and communication is essential for communication between individuals and quick recall of staff [48]. It is crucial to have clear and effective communication channels between hospital staff, emergency services, external communications with EMS agencies and local health departments for coordination in response, patient distribution, updating the status of the incident, and other entities involved in the crisis response process [48]. Additionally, having a rapid system for staff recall and mobilization is necessary for being present at the required locations. Hospitals can use various methods for internal communication and information dissemination among staff, including simultaneous use of public address systems, text messages, WhatsApp, and other social media. Moreover, radio communication can serve as a backup system in internal emergencies, so employee training for its use and communication is essential [48]. As hospitals may lose their primary communication channels, which are mainly telephone lines, during critical incidents [49], hospital managers should consider that communication challenges must not be limited to a single hospital, but rather may extend to regional and national mobile services, leading to difficulties in communicating with other healthcare facilities, emergency medical services, and other entities and agencies involved in crisis management [49]. Hospitals have used television and radio broadcasts to obtain information about the number and types of casualties in the incident, road conditions, and the status of other hospitals [50]. The hospital preparedness in this study was desirable in the domain of communication and information system. Although this level has been evaluated as average in most domestic studies [9, 51]. Therefore, to be prepared in the field of communication and information, all communication and information paths must be considered and included in the plan for responding to MICs.

The preparedness level of the surveyed hospitals in terms of equipment and medical supplies was desirable, which was similar to other studies conducted domestically [9, 34] and abroad [52, 53]. However, in a recent study conducted in Saudi Arabia on hospital preparedness evaluation, this level was not desirable [54]. The highest score in the infrastructure, equipment, and medical supplies domain was related to one of the hospitals in Sanandaj city. In addition, analytical results indicated a significant positive correlation between hospital preparedness for RTAs-MC and the number of active and approved hospital beds and hospital specialization in terms of infrastructure, equipment, and medical supplies. In fact, the hospital preparedness for RTAs-MC varies according to the hospital level [55, 33], and hospitals with higher levels of specialization that provide higher levels of care have better equipment, medical supplies, and infrastructure [56]. Other studies have also mentioned reasons for HDP, especially preparedness for RTAs-MC, such as having infrastructure and medical equipment, such as hospital beds, or infrastructure such as suitable space and facilities for treatment and care of casualties [9, 57]. The study emphasizes that hospitals with more beds and specialized services exhibit higher preparedness for road traffic accidents with mass casualties (RTAs-MC). This connection highlights the need to focus on hospital types and their capabilities in preparedness planning. Understanding that specialized hospitals with better resources play a crucial role in effective response, targeted interventions can enhance overall hospital readiness for RTAs-MC, ensuring they are well-equipped to handle such incidents.

Surge capacity refers to a hospital’s ability and resources to manage and provide services to a large number of patients and casualties in crisis situations [58], and it is one of the fundamental components for measuring HDP [59]. In a study conducted by Kaji et al. in hospitals in Los Angeles, it was reported that the surge capacity domain’s preparedness was severely limited [53]. However, the results of the present study also indicate desirable preparedness of hospitals in Kurdistan province in the surge capacity domain and a significant positive correlation between this domain and the number of ambulances and staff. The study results showed that only one hospital in Kurdistan province has a trauma center, and most hospitals in the province do not have emergency medicine specialists. Increasing hospital beds [59] and discharging low-risk patients [58] have been identified as key components of surge capacity. However, surge planning includes four essential elements (4 S): [1] staff, including medical and non-medical personnel; [2] stuff such as medical devices and ambulances; [3] structures or spaces that need to be modified as treatment areas or shelters; and [4] systems that refer to practical or mutual guidelines [60]. Therefore, to increase hospital preparedness in surge capacity domain, it is necessary to plan and take necessary actions to increase the above-mentioned elements.

In the present study, the preparedness level of the investigated hospitals in terms of triage and medical services was satisfactory (74.61). In contrast, in the study by Yousefian et al., this level was reported as average [9]. Triage is one of the key and effective principles for managing large-scale crises [29]. This principle provides the possibility of providing appropriate healthcare and ensuring survival for the injured in MCIs [61]. In fact, the high number of patients and limited resources during these types of incidents indicate the importance and necessity of triage. Additionally, performing triage in such incidents requires trained and skilled staff, as well as sufficient equipment [48], and should be based on standard guidelines and protocols. The simplicity and non-complexity of triage increase the speed of work and help predict the injured or patient’s condition to the best level [29]. Therefore, improving hospital preparedness for RTAs-MC is dependent on having an appropriate plan for triage and providing medical services, as disruption in triage performance and patient classification can lead to waste of resources and numerous consequences for the injured or patients, which may even endanger their lives [61].

Similar to other studies, the present study reported the preparedness level of hospitals in terms of human resources as average [9, 47]. Furthermore, the analytical results of the study revealed a significant and positive relationship between human resources and overall hospital preparedness for RTAs, as well as in the domains of command and control, capacity building, and specialist emergency medicine with infrastructure, equipment, medical supplies, and human resources. Human resources play a crucial role in HDP [6, 9]. Having professional, experienced, active, and up-to-date teams at the incident scene is one of the key aspects of ensuring the quality of service delivery and protecting healthcare facilities [7, 62]. On the other hand, hospital command and control based on the HICS, by providing services in the shortest possible time [13], preventing duplication of work [32], and regularly deploying human resources [44], enables individuals to take necessary responsibilities within the specified timeframe [49]. This, ultimately leading to effective management of human resources and provision of sufficient and efficient human resources [32].

While weakness in collaboration and coordination has been identified as one of the major problems in hospital crisis management, and its consequences may even exceed the lack of resources [63, 64], the preparedness level of hospitals in the area of collaboration and coordination in the present study was highly satisfactory (98.75) and obtained the highest score among the investigated domains in Kurdistan province. This finding is consistent with the study by Yousefian et al., which reported high preparedness levels in this domain for some hospitals in Iran (71.15) [9]. Creating a framework for coordination and communication between hospitals and other response organizations or authorities such as the police, Red Crescent, fire department, and coordinating with all hospital departments, pre-hospital emergency services, and other relevant health system sectors are the principles of hospital disaster management that have a significant role in enhancing HDP. Therefore, it is recommended to establish a logical management structure using the HICS to improve hospitals’ preparedness in this area, in which communication equipment, collaboration methods, common language, communication lines, and reporting channels are specified and provided [46, 63, 64].

The establishment and evaluation of safety and security protocols for disasters and emergencies are crucial aspects of HDP, and it is essential to ensure that these protocols meet the required standards and undergo regular testing [52]. Patient and staff safety is critical for continued healthcare service delivery during emergencies [65] and responding effectively to MCIs [52]. However, large-scale disasters and emergencies can significantly reduce hospital safety due to increased demand for services and their impact on hospital performance capacity [66]. Therefore, developing safety and security plans is a key component of hospital response plan [67]. The present study found that hospitals in Kurdistan province had a desirable level of preparedness in terms of safety and security, which was higher than that reported in previous studies, but some domestic studies [51], including the study by Ardalan et al. in 2014 [11], and some international studies [52], have reported inadequate safety in investigated hospitals. Therefore, hospital preparedness in terms of safety and security should be prioritized and included in their preparedness and response plan, and staff awareness in this area should be increased through training programs.

In the present study, the lowest level of preparedness was related to the domain of training and exercises (54.00), which was consistent with the study by Yousefian et al. in Iran [9]. However, a study in Lebanon showed that training courses and exercises were held in most of the investigated hospitals (95.8%) [68]. This finding indicates insufficient training and the lack of exercises or drills to respond to RTAs among hospital staff in the present study and similar studies in Iran, while improving hospital readiness to respond to such incidents requires planned exercises and drills [69] in coordination with other responsible organizations. These training courses and exercises should cover all procedures to enhance a quick and efficient response [68]. In addition, to improve the level of hospital preparedness, it is necessary to provide hospital preparedness program training to the targeted staff, and planning for staff training should be part of the hospital response plan [70].

## Limitations

Since this study was conducted in a number of hospitals in Kurdistan province, with specific topography and geographical location, the results cannot be generalized to other hospitals in Iran. Furthermore, the closeness of the study period to the COVID-19 crisis and the limitation of the measurement item with a response range of “Yes,” “Somewhat,” and “No” restricted the responses with more detailed answers to the questions. Therefore, it is recommended that the study be repeated at different times or with the utilization of other measurement methods such as in-depth interviews, qualitative analysis and document review.

## Conclusion

In conclusion, the study underscores the intricate nature of hospital preparedness for responding to Road Traffic Accidents with Mass Casualties (RTAs-MC), which is shaped by diverse influencing factors. To effectively address the challenges posed by RTAs-MC, specific recommendations are paramount. Hospitals need to prioritize investments in specialized infrastructure and medical equipment. The development of a comprehensive hospital response plan must carefully consider factors such as the hospital’s level of specialty, economic, cultural, geographical, and topographic features of the hospital location.

Moreover, the establishment of the Hospital Incident Command System (HICS), along with communication channels, is crucial. Ensuring information transfer before a crisis occurs enhances the overall responsiveness of hospitals. Future-oriented planning should actively engage all hospital staff, collaborating with other health sector departments, organizations, and non-health sector agencies responsible for RTAs-MC response. Ongoing efforts, including exercises, drills, lessons learned from real crises, and targeted training courses, are essential components in building the knowledge, awareness, and skills of hospital staff.

By implementing these recommendations, hospitals can significantly enhance their preparedness for RTAs-MC, contributing to a more resilient healthcare system capable of effectively addressing and mitigating the impact of such incidents.”

### Electronic supplementary material

Below is the link to the electronic supplementary material.

Supplementary Material 1

## Data Availability

The corresponding author can provide the datasets utilized and/or examined during the present study upon a reasonable request.

## Declarations

All experiments were performed in accordance with relevant guidelines and regulations related to the Declaration of Helsinki.

## Abbreviations

RTAs: Road traffic accidents
RTAs-MC: Road traffic accidents with mass casualties
HICS: Hospital incident command system
WHO: World Health Organization
HIS: Hospital Safety Index
FHSI: Farsi Hospital Safety Index
GEH: Government Educational Hospital
GGH: Government General Hospital
SSH: Social Security Hospital
MH: Military Hospital
PH: Private Hospital
MCIs: Mass Casualty Incidents
HDP: Hospital Disaster Preparedness
IRB: Institutional Review Board
