# Supplementary material for: Hospital preparedness assessment for road traffic accidents with mass casualties: a cross-sectional study in Kurdistan Province, Iran
Source: BMC Emerg Med. 2024 Apr 23;24:68. doi: 10.1186/s12873-024-00981-4 (PMC11036739; doi:10.1186/s12873-024-00981-4)
Supplement: Supplementary file 1 — Supplementary Material 1 [file 12873_2024_981_MOESM1_ESM.pdf]

Hospital Preparedness Checklist for Road Traffic Accidents with Mass Casualties (RTAs-MC)				
**Row**	**Item**	**Yes**	**Somewhat**	**No**
**Command and control**				
1	Are the plans and guidelines considering the hospital's response to RTIs with mass casualties?			
2	Are financial sources allocated to these plans?			
3	Are these plans and guidelines followed in all wards?			
4	Are plans reviews, exercises, revisions, and updating regularly performed?			
5	Is an ICS specified in the hospital?			
6	Are representatives of the following departments members of the ICS:<br>Hospital management, nursing management, laboratory, security, drug store, infection control, nutrition, facilities, services, and administrative and personnel affairs?			
7	Are the tasks and responsibilities of the members of ICS specified and notified?			
8	Have the members of ICS received the necessary training with regard to their role and status?			
9	Is the activation of ICS practiced at least twice a year?			
**Infrastructure and medical equipment**<br>Are the available infrastructures required for responding at the time of RTIs?				
1	Trauma room			
2	Triage room			
3	Trauma ICU or ATLS			
4	Burns room			
5	Laboratory			
6	Isolation room for injured people with communicable or infectious disease			
7	Helipad			
8	Hospital ambulance for inter-hospital deployment and transporting			
9	Parking space for pre-hospital emergency ambulances			
10	Appropriate location for the one-way entrance/exit of ambulances			
11	Enough space in terms of bed occupancy in the emergency department			
12	Are the maintenance, safekeeping, safety, replacement of equipment and medications performed based on guidelines?			
13	Is there sufficient equipment for personal protection and safety of personnel?<br>This includes gloves, face shields, goggles, disposable blades, and the proper disposal of biohazard waste.			
14	Are sufficient blood service supplies and equipment (e.g. blood set and blood warming pump) available?			

15	Are safety, maintenance, and monitoring of blood products and equipment performed based on guidelines?			
**Information and communication systems**				
1	Are communication services, including telephones, cell phones, radio, and satellite phones available in sufficient numbers?			
2	Are there standard messages and codes for paging and notifying red alerts and white alerts to personnel?			
3	Is the one in charge of sending red and white alerts known?			
4	Is the hospital spokesman trained for notifying the public of incidents?			
5	Is there an information agreement between the hospital and other involved organizations?			
6	Does the medical information system (MIS) record RTIs?			
7	Are the data related to RTIs managed, analyzed, and disseminated?			
8	Is the trauma mortality committee formed in the hospital?			
**Surge capacity**				
1	Is the maximum capacity of the hospital calculated for accepting patients (number of beds, human resources, essential resources, equipment, and tools)?			
2	Are there appropriate guidelines or policies for increasing hospital capacity?			
3	Early patient discharge			
4	Canceling elective and unnecessary surgeries			
5	Calculating maximum hospital capacity			
6	Estimating the increase in demand/patient reception			
7	Existence of a model for increasing hospital capacity			
8	Transferring patients between hospital wards/units			
9	Compatibility and proportion between reception and discharge			
10	Temporary use of units such as stagnant archives			
11	Home care for superficial injuries			
12	Are the required capacities (ventilators, incubators, and so on) available for vital life support and necessary cares for patients during transfer from the site of accident to the hospital?			
**Triage and medical services**				
1	Is there a standard and appropriate triage system?			
2	Are there guidelines for triage in RTIs with mass casualties?			
3	Is sufficient training on triage in RTIs with mass casualties given to the personnel?			
4	Is an experienced triaging official (e.g. A doctor of emergency or medicine or trauma or a trained and skillful emergency nurse as a supervisor) assigned for supervising the triaging process?			
5	Are sufficient triage labels available?			

6	Are entrance/exit routes to triage and waiting areas specified?			
7	Are entrance/exit routes to triage and waiting areas in appropriate conditions in terms of space, lighting, and safety?			
8	Is the triage area in the vicinity of key units (e.g. surgery, emergency, and intensive care)?			
9	Are there guidelines on decontamination in RTIs accompanied by chemical incidents?			
10	Is there an appropriate waiting area specified for injured patients and those who cannot move?			
11	Is a temporary morgue specified for keeping and transferring the bodies in regional RTIs with mass casualties?			
12	Are there sufficient dead body covers?			
13	Are there guidelines or policies for identifying casualties?			
**Safety and security**				
1	Are the ambulance entrance/exit routes specified?			
2	Are measures taken to control the crowd and traffic around and inside the hospital, triaging area, emergency department, and wards?			
3	Are key areas, for example, triage, treatment, and disinfection areas, pre-specified?			
4	Are there security personnel and guards in sufficient numbers?			
5	Is there an agreement with the police department or other security organizations when needed?			
**Human resources management**				
1	Is the emergency team sufficiently prepared for dealing with RTIs with mass casualties?			
2	Is a house physician specializing in emergency medicine present in the hospital?			
3	Are minimum needs of medical service providers and other personnel identified and satisfied for ensuring their effective performance in incidents?			
4	Are personnel welfare measures (e.g. commuting, child care, care at the time of illness, and disability of the personnel and their family members) taken?			
5	Are measures taken for the stress management of personnel, especially personnel of the emergency department?			
6	Are shift planning and rotation performed efficiently in order to reduce medical errors?			
7	Is a vaccination plan (against tetanus, hepatitis, and so on) planned for the emergency team in traumas?			
8	Is there a plan for providing and using extra and volunteer forces at the time of accidents?			
9	Is in-service training and practice seriously pursued for the personnel of the operating room, emergency department, and intensive care units in order to enhance hospital capacity and efficiency?			

**Coordination and cooperation**				
1	Are MOA signed with other organizations, for example, fire department, blood transfusion organization, and the police department, in order to provide medical services in RTIs with mass casualties?			
2	Are agreements signed with other hospitals in order to provide medical services in RTIs with mass casualties?			
**Training and exercise**				
1	Are official training programs held for personnel about RTIs with mass casualties and trauma care?			
2	Is the necessary training provided for personnel on their role and responsibilities in RTIs with mass casualties?			
3	Are theoretical and practical exercises performed for efficiently responding to RTIs with mass casualties (at least twice a year)?			
4	Are exercises performed in association with other involved organizations?			
5	Are there trauma-related quality improvement programs available?			
